# The Use of Purgatives in Abdominal Operations

**Published:** 1904-05-28

**Authors:** 


### THE USE OF PURGATIVES IN ABDOMINAL OPERATIONS.

One of the many bugbears of abdominal surgery is the intestinal paralysis which is often such a frequent source of trouble in the next few days after operation. The usual methods of coping with this complication are the use of drugs, *e.g.*, strychnine and peppermint, by the mouth, rectal enemata containing turpentine or asafœtida, slightly changing the position of the patient in bed, and so forth. But with all these means the tympanites may persist and even lead to the patient's death. A suggestion put forward by Mr. F. Barrington[1] is therefore well worthy of serious consideration. He recommends the routine administration of a purgative shortly before operation. For the last four years he has been in the habit of giving his patients five grains of calomel one hour and a half before operation, with satisfactory results. In septic cases two drachms of magnesium sulphate in concentrated solution, with lemon juice, are given in addition immediately before the patient is taken into the operating theatre. There are two reasons for this treatment. Mr. Barrington claims that the peristalsis caused is of value not only because it obviates the development of tympanites, but also because it produces intra-peritoneal currents which help in the removal of micro-organisms from the peritoneal cavity into the lymphatics. The movement also tends to prevent the formation of post-operative adhesions.

[1] Aust. Med. Gaz., February.

